# Supplementary material for: Pyruvate Dehydrogenase Kinase 1 inhibition mediated oxidative phosphorylation enhancement in cartilage promotes osteoarthritis progression
Source: BMC Musculoskelet Disord. 2023 Jul 20;24:597. doi: 10.1186/s12891-023-06585-6 (PMC10357736; doi:10.1186/s12891-023-06585-6)
Supplement: Supplementary file 1 — Additional file 1. [file 12891_2023_6585_MOESM1_ESM.zip › MF.Gsea.1653627316242/GOMF_EXOPEPTIDASE_ACTIVITY.html]

Details for gene set GOMF\_EXOPEPTIDASE\_ACTIVITY[GSEA]

|  || Dataset | 55457\_collapsed\_to\_symbols.cls.cls #OA\_versus\_normal.cls.cls #OA\_versus\_normal\_repos |
| Phenotype | cls.cls#OA\_versus\_normal\_repos |
| Upregulated in class | OA |
| GeneSet | GOMF\_EXOPEPTIDASE\_ACTIVITY |
| Enrichment Score (ES) | 0.30213508 |
| Normalized Enrichment Score (NES) | 1.3525827 |
| Nominal p-value | 0.043010753 |
| FDR q-value | 0.36857215 |
| FWER p-Value | 1.0 |
Table: GSEA Results Summary

  

Fig 1: Enrichment plot: GOMF\_EXOPEPTIDASE\_ACTIVITY      
 Profile of the Running ES Score & Positions of GeneSet Members on the Rank Ordered List

  

| SYMBOL | TITLE | RANK IN GENE LIST | RANK METRIC SCORE | RUNNING ES | CORE ENRICHMENT || 1 | LTA4H | leukotriene A4 hydrolase [Source:HGNC Symbol;Acc:HGNC:6710] | 18 | 1.114 | 0.0406 | Yes |
| 2 | SCRN3 | secernin 3 [Source:HGNC Symbol;Acc:HGNC:30382] | 155 | 0.855 | 0.0622 | Yes |
| 3 | PEPD | peptidase D [Source:HGNC Symbol;Acc:HGNC:8840] | 167 | 0.842 | 0.0931 | Yes |
| 4 | ERAP2 | endoplasmic reticulum aminopeptidase 2 [Source:HGNC Symbol;Acc:HGNC:29499] | 212 | 0.811 | 0.1202 | Yes |
| 5 | CTSC | cathepsin C [Source:HGNC Symbol;Acc:HGNC:2528] | 227 | 0.801 | 0.1493 | Yes |
| 6 | CPQ | carboxypeptidase Q [Source:HGNC Symbol;Acc:HGNC:16910] | 257 | 0.780 | 0.1764 | Yes |
| 7 | ZMPSTE24 | zinc metallopeptidase STE24 [Source:HGNC Symbol;Acc:HGNC:12877] | 451 | 0.682 | 0.1871 | Yes |
| 8 | CPVL | carboxypeptidase vitellogenic like [Source:HGNC Symbol;Acc:HGNC:14399] | 459 | 0.677 | 0.2121 | Yes |
| 9 | AGBL3 | AGBL carboxypeptidase 3 [Source:HGNC Symbol;Acc:HGNC:27981] | 593 | 0.626 | 0.2253 | Yes |
| 10 | ERAP1 | endoplasmic reticulum aminopeptidase 1 [Source:HGNC Symbol;Acc:HGNC:18173] | 634 | 0.610 | 0.2452 | Yes |
| 11 | FAP | fibroblast activation protein alpha [Source:HGNC Symbol;Acc:HGNC:3590] | 756 | 0.580 | 0.2576 | Yes |
| 12 | MINDY2 | MINDY lysine 48 deubiquitinase 2 [Source:HGNC Symbol;Acc:HGNC:26954] | 863 | 0.552 | 0.2701 | Yes |
| 13 | CPA3 | carboxypeptidase A3 [Source:HGNC Symbol;Acc:HGNC:2298] | 1259 | 0.465 | 0.2568 | Yes |
| 14 | CNDP2 | carnosine dipeptidase 2 [Source:HGNC Symbol;Acc:HGNC:24437] | 1476 | 0.432 | 0.2563 | Yes |
| 15 | SCPEP1 | serine carboxypeptidase 1 [Source:HGNC Symbol;Acc:HGNC:29507] | 1520 | 0.426 | 0.2690 | Yes |
| 16 | AOPEP | aminopeptidase O (putative) [Source:HGNC Symbol;Acc:HGNC:1361] | 1740 | 0.393 | 0.2667 | Yes |
| 17 | CPE | carboxypeptidase E [Source:HGNC Symbol;Acc:HGNC:2303] | 1796 | 0.385 | 0.2769 | Yes |
| 18 | CTSH | cathepsin H [Source:HGNC Symbol;Acc:HGNC:2535] | 1803 | 0.384 | 0.2909 | Yes |
| 19 | CPB1 | carboxypeptidase B1 [Source:HGNC Symbol;Acc:HGNC:2299] | 1918 | 0.370 | 0.2960 | Yes |
| 20 | CTSA | cathepsin A [Source:HGNC Symbol;Acc:HGNC:9251] | 2014 | 0.360 | 0.3021 | Yes |
| 21 | GGH | gamma-glutamyl hydrolase [Source:HGNC Symbol;Acc:HGNC:4248] | 2284 | 0.328 | 0.2935 | No |
| 22 | KDM8 | lysine demethylase 8 [Source:HGNC Symbol;Acc:HGNC:25840] | 2594 | 0.291 | 0.2804 | No |
| 23 | AGBL5 | AGBL carboxypeptidase 5 [Source:HGNC Symbol;Acc:HGNC:26147] | 2613 | 0.290 | 0.2899 | No |
| 24 | ERMP1 | endoplasmic reticulum metallopeptidase 1 [Source:HGNC Symbol;Acc:HGNC:23703] | 2647 | 0.286 | 0.2981 | No |
| 25 | TRHDE | thyrotropin releasing hormone degrading enzyme [Source:HGNC Symbol;Acc:HGNC:30748] | 2755 | 0.275 | 0.3001 | No |
| 26 | LAP3 | leucine aminopeptidase 3 [Source:HGNC Symbol;Acc:HGNC:18449] | 3044 | 0.245 | 0.2869 | No |
| 27 | TPP1 | tripeptidyl peptidase 1 [Source:HGNC Symbol;Acc:HGNC:2073] | 3735 | 0.182 | 0.2399 | No |
| 28 | DPP3 | dipeptidyl peptidase 3 [Source:HGNC Symbol;Acc:HGNC:3008] | 3822 | 0.174 | 0.2398 | No |
| 29 | CPZ | carboxypeptidase Z [Source:HGNC Symbol;Acc:HGNC:2333] | 4043 | 0.152 | 0.2283 | No |
| 30 | HPN | hepsin [Source:HGNC Symbol;Acc:HGNC:5155] | 4185 | 0.139 | 0.2226 | No |
| 31 | VASH2 | vasohibin 2 [Source:HGNC Symbol;Acc:HGNC:25723] | 4345 | 0.124 | 0.2149 | No |
| 32 | DPP8 | dipeptidyl peptidase 8 [Source:HGNC Symbol;Acc:HGNC:16490] | 4571 | 0.107 | 0.2014 | No |
| 33 | NAALAD2 | N-acetylated alpha-linked acidic dipeptidase 2 [Source:HGNC Symbol;Acc:HGNC:14526] | 4870 | 0.082 | 0.1812 | No |
| 34 | DPEP3 | dipeptidase 3 [Source:HGNC Symbol;Acc:HGNC:23029] | 5176 | 0.058 | 0.1596 | No |
| 35 | CPB2 | carboxypeptidase B2 [Source:HGNC Symbol;Acc:HGNC:2300] | 5279 | 0.050 | 0.1535 | No |
| 36 | XPNPEP1 | X-prolyl aminopeptidase 1 [Source:HGNC Symbol;Acc:HGNC:12822] | 5620 | 0.022 | 0.1278 | No |
| 37 | QPCT | glutaminyl-peptide cyclotransferase [Source:HGNC Symbol;Acc:HGNC:9753] | 5698 | 0.016 | 0.1224 | No |
| 38 | F11 | coagulation factor XI [Source:HGNC Symbol;Acc:HGNC:3529] | 5767 | 0.010 | 0.1175 | No |
| 39 | NAALADL1 | N-acetylated alpha-linked acidic dipeptidase like 1 [Source:HGNC Symbol;Acc:HGNC:23536] | 6024 | -0.006 | 0.0978 | No |
| 40 | MINDY1 | MINDY lysine 48 deubiquitinase 1 [Source:HGNC Symbol;Acc:HGNC:25648] | 6126 | -0.014 | 0.0905 | No |
| 41 | CTSZ | cathepsin Z [Source:HGNC Symbol;Acc:HGNC:2547] | 6292 | -0.027 | 0.0786 | No |
| 42 | PRSS16 | serine protease 16 [Source:HGNC Symbol;Acc:HGNC:9480] | 6341 | -0.031 | 0.0760 | No |
| 43 | METAP1 | methionyl aminopeptidase 1 [Source:HGNC Symbol;Acc:HGNC:15789] | 6543 | -0.045 | 0.0620 | No |
| 44 | PRCP | prolylcarboxypeptidase [Source:HGNC Symbol;Acc:HGNC:9344] | 6725 | -0.057 | 0.0501 | No |
| 45 | AEBP1 | AE binding protein 1 [Source:HGNC Symbol;Acc:HGNC:303] | 6822 | -0.065 | 0.0450 | No |
| 46 | ACE2 | angiotensin converting enzyme 2 [Source:HGNC Symbol;Acc:HGNC:13557] | 6951 | -0.076 | 0.0379 | No |
| 47 | FOLH1 | folate hydrolase 1 [Source:HGNC Symbol;Acc:HGNC:3788] | 7048 | -0.083 | 0.0335 | No |
| 48 | DPP4 | dipeptidyl peptidase 4 [Source:HGNC Symbol;Acc:HGNC:3009] | 7132 | -0.091 | 0.0305 | No |
| 49 | NCLN | nicalin [Source:HGNC Symbol;Acc:HGNC:26923] | 7259 | -0.100 | 0.0244 | No |
| 50 | CPD | carboxypeptidase D [Source:HGNC Symbol;Acc:HGNC:2301] | 7433 | -0.114 | 0.0152 | No |
| 51 | RNPEPL1 | arginyl aminopeptidase like 1 [Source:HGNC Symbol;Acc:HGNC:10079] | 8006 | -0.155 | -0.0236 | No |
| 52 | DPP6 | dipeptidyl peptidase like 6 [Source:HGNC Symbol;Acc:HGNC:3010] | 8027 | -0.158 | -0.0192 | No |
| 53 | DPEP2 | dipeptidase 2 [Source:HGNC Symbol;Acc:HGNC:23028] | 8189 | -0.170 | -0.0253 | No |
| 54 | CPM | carboxypeptidase M [Source:HGNC Symbol;Acc:HGNC:2311] | 8199 | -0.171 | -0.0196 | No |
| 55 | BLMH | bleomycin hydrolase [Source:HGNC Symbol;Acc:HGNC:1059] | 8219 | -0.173 | -0.0146 | No |
| 56 | DNPEP | aspartyl aminopeptidase [Source:HGNC Symbol;Acc:HGNC:2981] | 8371 | -0.184 | -0.0194 | No |
| 57 | VASH1 | vasohibin 1 [Source:HGNC Symbol;Acc:HGNC:19964] | 8473 | -0.191 | -0.0201 | No |
| 58 | ENPEP | glutamyl aminopeptidase [Source:HGNC Symbol;Acc:HGNC:3355] | 8521 | -0.195 | -0.0164 | No |
| 59 | CPN1 | carboxypeptidase N subunit 1 [Source:HGNC Symbol;Acc:HGNC:2312] | 8613 | -0.202 | -0.0159 | No |
| 60 | RCE1 | Ras converting CAAX endopeptidase 1 [Source:HGNC Symbol;Acc:HGNC:13721] | 8867 | -0.222 | -0.0273 | No |
| 61 | XPNPEP2 | X-prolyl aminopeptidase 2 [Source:HGNC Symbol;Acc:HGNC:12823] | 8997 | -0.231 | -0.0286 | No |
| 62 | MME | membrane metalloendopeptidase [Source:HGNC Symbol;Acc:HGNC:7154] | 9287 | -0.255 | -0.0415 | No |
| 63 | DPEP1 | dipeptidase 1 [Source:HGNC Symbol;Acc:HGNC:3002] | 9353 | -0.260 | -0.0368 | No |
| 64 | MMP17 | matrix metallopeptidase 17 [Source:HGNC Symbol;Acc:HGNC:7163] | 9369 | -0.261 | -0.0281 | No |
| 65 | SCRN1 | secernin 1 [Source:HGNC Symbol;Acc:HGNC:22192] | 9391 | -0.263 | -0.0199 | No |
| 66 | AGBL2 | AGBL carboxypeptidase 2 [Source:HGNC Symbol;Acc:HGNC:26296] | 9559 | -0.279 | -0.0224 | No |
| 67 | ACE | angiotensin I converting enzyme [Source:HGNC Symbol;Acc:HGNC:2707] | 9720 | -0.294 | -0.0238 | No |
| 68 | CPA2 | carboxypeptidase A2 [Source:HGNC Symbol;Acc:HGNC:2297] | 9774 | -0.298 | -0.0167 | No |
| 69 | METAP2 | methionyl aminopeptidase 2 [Source:HGNC Symbol;Acc:HGNC:16672] | 9972 | -0.318 | -0.0201 | No |
| 70 | NPEPPS | aminopeptidase puromycin sensitive [Source:HGNC Symbol;Acc:HGNC:7900] | 10157 | -0.337 | -0.0217 | No |
| 71 | TFRC | transferrin receptor [Source:HGNC Symbol;Acc:HGNC:11763] | 10184 | -0.339 | -0.0110 | No |
| 72 | XPNPEP3 | X-prolyl aminopeptidase 3 [Source:HGNC Symbol;Acc:HGNC:28052] | 10353 | -0.356 | -0.0107 | No |
| 73 | LNPEP | leucyl and cystinyl aminopeptidase [Source:HGNC Symbol;Acc:HGNC:6656] | 10453 | -0.367 | -0.0045 | No |
| 74 | AGTPBP1 | ATP/GTP binding carboxypeptidase 1 [Source:HGNC Symbol;Acc:HGNC:17258] | 10752 | -0.399 | -0.0128 | No |
| 75 | MMP15 | matrix metallopeptidase 15 [Source:HGNC Symbol;Acc:HGNC:7161] | 11003 | -0.430 | -0.0161 | No |
| 76 | NPEPL1 | aminopeptidase like 1 [Source:HGNC Symbol;Acc:HGNC:16244] | 11040 | -0.435 | -0.0025 | No |
| 77 | ANPEP | "alanyl aminopeptidase, membrane [Source:HGNC Symbol;Acc:HGNC:500]" | 11404 | -0.487 | -0.0124 | No |
| 78 | TPP2 | tripeptidyl peptidase 2 [Source:HGNC Symbol;Acc:HGNC:12016] | 11492 | -0.500 | -0.0003 | No |
| 79 | CPA1 | carboxypeptidase A1 [Source:HGNC Symbol;Acc:HGNC:2296] | 11581 | -0.512 | 0.0121 | No |
| 80 | TFR2 | transferrin receptor 2 [Source:HGNC Symbol;Acc:HGNC:11762] | 11750 | -0.541 | 0.0194 | No |
| 81 | MMP16 | matrix metallopeptidase 16 [Source:HGNC Symbol;Acc:HGNC:7162] | 11819 | -0.553 | 0.0349 | No |
| 82 | QPCTL | glutaminyl-peptide cyclotransferase like [Source:HGNC Symbol;Acc:HGNC:25952] | 12163 | -0.634 | 0.0321 | No |
| 83 | MMP14 | matrix metallopeptidase 14 [Source:HGNC Symbol;Acc:HGNC:7160] | 12286 | -0.672 | 0.0479 | No |
Table: GSEA details [plain text format]

  

Fig 2: GOMF\_EXOPEPTIDASE\_ACTIVITY      
 Blue-Pink O' Gram in the Space of the Analyzed GeneSet

  

Fig 3: GOMF\_EXOPEPTIDASE\_ACTIVITY: Random ES distribution      
 Gene set null distribution of ES for **GOMF\_EXOPEPTIDASE\_ACTIVITY**
